# Graph Convolutional Networks Reveal Network-Level Functional Dysconnectivity in Schizophrenia

**DOI:** 10.1093/schbul/sbac047

**Published:** 2022-05-15

**Authors:** Du Lei, Kun Qin, Walter H L Pinaya, Jonathan Young, Therese Van Amelsvoort, Machteld Marcelis, Gary Donohoe, David O Mothersill, Aiden Corvin, Sandra Vieira, Su Lui, Cristina Scarpazza, Celso Arango, Ed Bullmore, Qiyong Gong, Philip McGuire, Andrea Mechelli

**Affiliations:** Huaxi MR Research Center (HMRRC), Department of Radiology, West China Hospital, Sichuan University, Chengdu, China; Department of Psychosis Studies, Institute of Psychiatry, Psychology & Neuroscience, King’s College London, London, UK; Department of Psychiatry and Behavioral Neuroscience, University of Cincinnati College of Medicine, Cincinnati, OH, USA; Huaxi MR Research Center (HMRRC), Department of Radiology, West China Hospital, Sichuan University, Chengdu, China; Department of Psychiatry and Behavioral Neuroscience, University of Cincinnati College of Medicine, Cincinnati, OH, USA; Department of Biomedical Engineering, School of Biomedical Engineering & Imaging Sciences, King’s College London, London, UK; Department of Neuroimaging, Institute of Psychiatry, Psychology, and Neuroscience, King’s College London, London, UK; Department of Psychiatry and Neuropsychology, School for Mental Health and Neuroscience, Maastricht University Medical Center, Maastricht, The Netherlands; Department of Psychiatry and Neuropsychology, School for Mental Health and Neuroscience, Maastricht University Medical Center, Maastricht, The Netherlands; Mental Health Care Institute Eindhoven (GGzE), Eindhoven, The Netherlands; School of Psychology & Center for Neuroimaging and Cognitive Genomics, NUI Galway University, Galway, Ireland; Psychology Department, School of Business, National College of Ireland, Dublin, Ireland; Department of Psychiatry, School of Medicine, Trinity College Dublin, Dublin, Ireland; Department of Psychosis Studies, Institute of Psychiatry, Psychology & Neuroscience, King’s College London, London, UK; Huaxi MR Research Center (HMRRC), Department of Radiology, West China Hospital, Sichuan University, Chengdu, China; Department of Psychosis Studies, Institute of Psychiatry, Psychology & Neuroscience, King’s College London, London, UK; Department of General Psychology, University of Padova, Padova, Italy; Padova Neuroscience Centre, University of Padova, Padova, Italy; Institute of Psychiatry and Mental Health, Department of Child and Adolescent Psychiatry, Hospital General Universitario Gregorio Marañon, School of Medicine, Universidad Complutense Madrid, IiSGM, CIBERSAM, Madrid, Spain; Brain Mapping Unit, Department of Psychiatry, University of Cambridge, Cambridge, UK; Huaxi MR Research Center (HMRRC), Department of Radiology, West China Hospital, Sichuan University, Chengdu, China; Research Unit of Psychoradiology, Chinese Academy of Medical Sciences, Chengdu, Sichuan, China; Department of Radiology, West China Xiamen Hospital of Sichuan University, Xiamen, Fujian, China; Department of Psychosis Studies, Institute of Psychiatry, Psychology & Neuroscience, King’s College London, London, UK; Department of Psychosis Studies, Institute of Psychiatry, Psychology & Neuroscience, King’s College London, London, UK

**Keywords:** neuroimaging, psychosis, machine learning, connectome, graph analysis, magnetic resonance imaging

## Abstract

**Background and Hypothesis:**

Schizophrenia is increasingly understood as a disorder of brain dysconnectivity. Recently, graph-based approaches such as graph convolutional network (GCN) have been leveraged to explore complex pairwise similarities in imaging features among brain regions, which can reveal abstract and complex relationships within brain networks.

**Study Design:**

We used GCN to investigate topological abnormalities of functional brain networks in schizophrenia. Resting-state functional magnetic resonance imaging data were acquired from 505 individuals with schizophrenia and 907 controls across 6 sites. Whole-brain functional connectivity matrix was extracted for each individual. We examined the performance of GCN relative to support vector machine (SVM), extracted the most salient regions contributing to both classification models, investigated the topological profiles of identified salient regions, and explored correlation between nodal topological properties of each salient region and severity of symptom.

**Study Results:**

GCN enabled nominally higher classification accuracy (85.8%) compared with SVM (80.9%). Based on the saliency map, the most discriminative brain regions were located in a distributed network including striatal areas (ie, putamen, pallidum, and caudate) and the amygdala. Significant differences in the nodal efficiency of bilateral putamen and pallidum between patients and controls and its correlations with negative symptoms were detected in post hoc analysis.

**Conclusions:**

The present study demonstrates that GCN allows classification of schizophrenia at the individual level with high accuracy, indicating a promising direction for detection of individual patients with schizophrenia. Functional topological deficits of striatal areas may represent a focal neural deficit of negative symptomatology in schizophrenia.

## Introduction

Schizophrenia is a severe mental disorder characterized by delusions, hallucinations, and disorganized thinking, which affects approximately 0.3%–0.7% of the world’s population.^1^ Given the complex and heterogeneous clinical presentation, diagnosis solely based on clinical observation may lack accuracy and objectivity.^2–4^ Therefore, there is an urgent need to establish reliable diagnostic biomarkers in order to develop personalized treatments within the precision medicine framework.

Previous research on imaging biomarkers has largely focused on single brain regions and localized connectivity.^5,6^ However, the human brain is a highly interconnected network, and the emergence of psychiatric illness is thought to be underpinned by a disruption of normal functional integration among cortical and subcortical regions.^7–11^ Over the past 2 decades, neuroimaging studies have identified widespread functional dysconnectivity in individuals with schizophrenia relative to controls.^12–18^ These findings have led to the conclusion that schizophrenia cannot be explained in terms of localized dysfunction within specific brain areas and is better understood as a disruption of network-level functional organization.^19–22^ The study of functional brain organization has the potential to identify predictive biomarkers for neurodevelopmental and neuropsychiatric disorders and shed light on their underlying mechanisms.

Recently, neuroimaging studies have employed machine-learning techniques that enable statistical inferences at the level of the individual patient.^23^ Notably, deep neural networks are capable of capturing subtle hidden representations in the data.^24^ However, while schizophrenia is increasingly understood as a disorder of brain dysconnectivity, most machine-learning models adopted in previous studies typically worked based on independent functional connections instead of the connectome itself.^25^ In contrast, the use of graphs provides an alternative approach to network-level analysis which does capture topological information within brain networks.^26^ In neuroscience, where such representations are commonly used to model structural or functional connectivity between a set of brain regions, graphs have proven to be of great importance. Thus, graph-based neural networks (GNNs) have recently gained significant attention,^27^ as this type of deep leaning techniques is able to directly deal with the non-Euclidean graph data structure (eg, social network, protein interaction network, and brain network), capturing and abstracting complex network-level information via the local features and neighborhood relationships. Recent studies applying GNN to brain networks in individuals with schizophrenia, have achieved promising results. For example, Chang et al found that, compared to support vector machine (SVM), a GNN model of electroencephalography-based brain networks showed better performance in distinguishing among first-episode, chronic schizophrenia patients and controls.^28^ Likewise, by using GNN combined with functional connectome data, Oh et al achieved a classification accuracy of 83.13%, which outperformed alternative machine-learning methods.^29^

One of the most widely used model in GNN family is the graph convolutional network (GCN). Motivated by convolutional neural network (CNN), GCN was designed to perform convolution operation on graph structure to aggregate local and neighboring information to generate new feature maps. The GCN has been successfully applied in previous publications to characterize autism spectrum disorder,^30^ Alzheimer’s disease,^31^ depression,^32^ and sex.^33^ However, most current application of GCN is limited to small dataset, and the model performance could be potentially unreliable. Moreover, in neuroimaging studies of brain disorders, the identification of the neural correlates of the disease under investigation is critical. In particular, locating brain areas with a critical role in the disruption of specific connections is among the most important goals in the study of the human connectome.^34^ By capturing brain development and disease patterns in the neuroimaging data, GCN may reveal clinically meaningful network-level functional dysconnectivity, which would be difficult to detect using traditional machine-learning methods. Therefore, the use of representative GCN classifiers on large-scale and multisite data can not only validate the feasibility of GNN methods for schizophrenia classification but also reveal reliable neurobiological underpinnings of schizophrenia at connectome level.

Here, we established a GCN model to compare patients with schizophrenia and controls using multisite neuroimaging data. We used 6 independent datasets resulting in a total sample of 505 patients with schizophrenia and 907 controls. In order to assess the reliability of the findings, we performed a separate analysis on each single dataset in addition to pooling all datasets together. Our first hypothesis was that using a GCN model would allow diagnostic classification with a higher level of accuracy than a widely used traditional machine-learning model (ie, SVM). In addition, previous studies have shown that spatially segregated salient regions can be identified in non-Euclidean space on GCN,^35^ making it possible to effectively map the most important brain regions for the task under consideration.^33^ Therefore, our second hypothesis was that GCN would provide distinct saliency maps of brain regions and show significant topological deficits related to clinical measures, reflecting the ability of this technique to capture network-level topological information, compared to SVM, which cannot capture abstract and complex relationships within networks.

## Methods

### Participants

A total of 1412 subjects comprising 505 patients with schizophrenia and 907 controls were included in our study (table 1). The diagnosis of schizophrenia was established using the Structured Clinical Interview for DSM-IV (SCID).^36^ Written informed consent was provided for all participants, and data collection was approved by the local Institutional Review Board at each site. Detailed information about eligibility criteria is presented in Supplementary Information.

**Table 1.** Demographic and Clinical Characteristics of Participants^a^

	Dataset 1 (*n* = 518)		Dataset 2 (*n* = 340)	Dataset 3 (*n* = 112)		Dataset 4 (*n* = 115)		Dataset 5 (*n* = 139)		Dataset 6 (*n* = 188)	
Variables	SCZ	CON	CON	SCZ	CON	SCZ	CON	SCZ	CON	SCZ	CON
Sample size	301	217	340	49	63	32	83	67	72	56	132
Disease stage	FE	—	—	EST^b^	—	EST	—	EST	—	EST	—
Age (y)	24.2 ± 8.5	33.80 ± 15.63	24.7 ± 9.0	29.0 ± 6.4	29.6 ± 10.6	40.9 ± 10.9	28.1 ± 9.0	38.3 ± 14.1	35.9 ± 11.7	36.2 ± 8.5	31.0 ± 8.6
Gender (M/F)	131/170	85/132	157/183	38/11	25/38	24/8	38/45	54/13	51/21	42/14	69/63
Handedness (R/L/B)	301/0/0	217/0/0	NA	40/7/2	54/7/2	32/0/0	83/0/0	55/10/2	69/1/2	NA	NA
Education (y)	12.1 ± 3.0	11.4 ± 3.4	NA	16.6 ± 2.0	17.4 ± 2.0	14.7 ± 4.4	17.7 ± 3.3	NA	NA	NA	NA
Medication (An/Dn)	0/90	NA	NA	48/1	NA	23/5^c^	NA	67/0	NA	45/4^g^	NA
PANSS total	76.5 ± 24.4^d^	NA	NA	44.2 ± 12.4	NA	NA	NA	58.8 ± 14.4^e^	NA	NA	NA
PANSS positive	20.2 ± 9.0^d^	NA	NA	10.1 ± 4.4	NA	NA	NA	14.4 ± 4.8^e^	NA	NA	NA
PANSS negative	17.2 ± 7.5^d^	NA	NA	10.8 ± 5.3	NA	NA	NA	15.0 ± 5.4^e^	NA	NA	NA
PANSS general	39.5 ± 12.9^d^	NA	NA	23.2 ± 5.5	NA	NA	NA	29.4 ± 8.6^e^	NA	NA	NA
SAPS	NA	NA	NA	NA	NA	7.6 ± 12.3^f^	NA	NA	NA	23.1 ± 17.0^h^	NA
SANS	NA	NA	NA	NA	NA	13.3 ± 17.9^f^	NA	NA	NA	28.3 ± 16.1^h^	NA

*Note*: An, antipsychotic medication; B, ambidextrous; CON, control; Dn, drug-naive; EST, established; F, female; FE, first episode; L, left; M, male; NA, not available; PANSS, Positive and Negative Syndrome Scale; R, right; SANS, Scale for the Assessment of Negative Symptoms; SAPS, Scale for the Assessment of Positive Symptoms; SCZ, schizophrenia.

^a^Data are presented as mean ± standard deviation.

^b^Patients were diagnosed with established schizophrenia if duration of illness was more than 24 months.

^c^Data available for 28 of 32 patients.

^d^Data available for 272 of 301 patients.

^e^Data available for 49 of 67 patients.

^f^Data available for 24 of 32 patients.

^g^Data available for 49 of 56 patients.

^h^Data available for 50 of 56 patients.

### Image Acquisition and Processing

Resting-state functional magnetic resonance imaging (rs-fMRI) scans were acquired using different scanners and acquisition parameters for each dataset. Detailed information about scanner and acquisition parameters is presented in Supplementary Information. To mitigate methodological heterogeneity across datasets, a unified image preprocessing pipeline was performed using Statistical Parametric Mapping 12 (SPM12) and Data Processing Assistant for Resting-State fMRI (DPARSF) software.^37^ Preprocessing steps included slice timing correction, head motion correction, normalization, and removal of nuisance confounds (for specific preprocessing procedures and parameters, see Supplementary Information). The preprocessed rs-fMRI scans were subsequently used to estimate the whole-brain functional connectivity networks. First, the whole brain was divided into 90 anatomical regions according to the Automatic Anatomical Labeling (AAL) atlas. Next, the functional connectivity matrix was estimated as Pearson’s correlation coefficients of the time series between all pairs of regions. Fisher *r*-to-*z* transformation was further applied to convert each correlation coefficient to *z*-score for normality.

### ComBat Harmonization

In the context of machine learning based on multisite neuroimaging data, the site effects derived from scanners and sequence parameters may be equivalent to or even more prominent than the actual case-control effect, considerably impairing the model performance. To remove this unwanted site effect and expose the actual functional abnormalities in patients, we used a known harmonization method called ComBat.^38^ The effects of ComBat harmonization were quantified to see how it works on our dataset. Detailed information can be found in Supplementary Information.

### Graph Convolutional Network

**Fig. 1.**
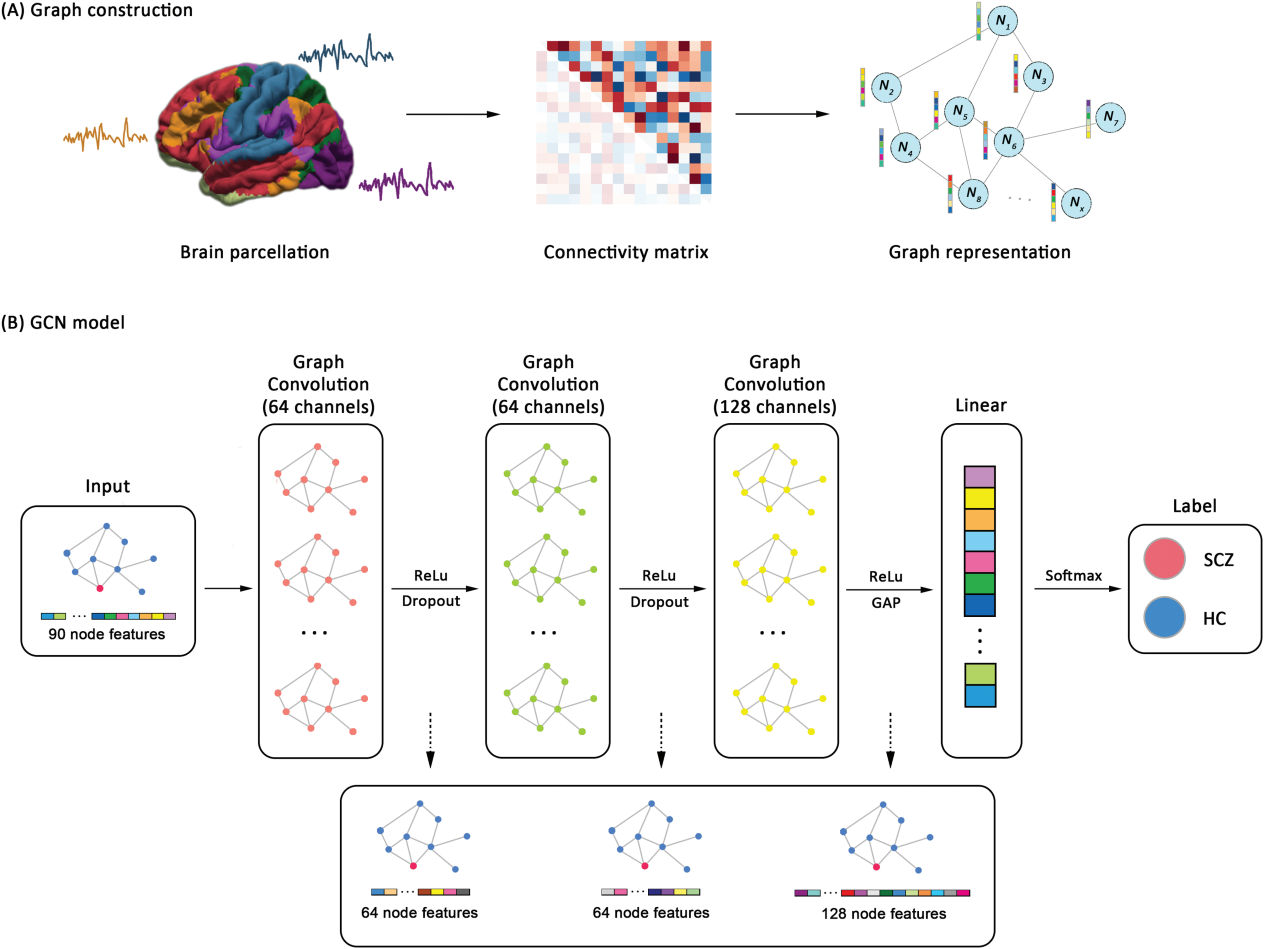
The overall pipeline of graph convolutional network model. (A) Graph construction for each individual using resting-state functional connectivity. (B) The architecture and implementation of graph convolutional network. *Note*: GAP, global average pooling; HC, healthy controls; ReLu, Rectified Linear Unit; SCZ, schizophrenia.

The GCN was used to perform single-subject classification of patients with schizophrenia and controls. The overall pipeline of our GCN model is shown in figure 1. Assuming a feature matrix $X\in R^{a\times b}$, where *a* is the number of nodes and *b* is the number of features per node, we typically encoded the feature matrix *X* into a weighted graph structure G=(V,E,W), where *V* and *E* were sets of nodes and edges, respectively, and $W\in R^{a\times a}$ was the weighted adjacency matrix. In our study, the whole-brain functional connectivity matrix was treated as individual feature and represented as a graph structure. Specifically, each brain region represented a node, and the corresponding node feature represented the functional connectivity between each region and all the other regions. The adjacency matrix was subsequently calculated via *K*-nearest neighbors (KNN) algorithm, which estimated the similarity determined by the Euclidean distance between pairs of nodes. This approach to graph modeling in GCN has achieved high levels of success in previous studies.^39^ We set the *k* value of the KNN algorithm as 10 based on the sensitivity analysis of dynamic *k* values (see Supplementary Information). The mathematics and hyperparameters of our GCN model are presented in Supplementary Information in detail.

We separately used pooled stratified cross-validation and leave-one-site-out (LOSO) cross-validation to split the samples into training and testing sets for the evaluation of GCN performance. For the LOSO cross-validation, each dataset was consecutively left as testing set during each loop, and the remaining datasets were used to train the model. Since dataset 2 only includes controls, we failed to include this dataset to test the model performance. For the pooled stratified cross-validation, the pooled samples were randomly divided into 10-folds, of which 1-fold served as testing set, and the remaining 9-folds were used as the training set. This strategy guarantees that training and testing sets contain the equivalent proportion of each class. Because the ComBat harmonization was applied to remove site effect, we did not balance the proportion of each site in the training and testing sets. The model performance based on the testing set was further assessed in terms of balanced accuracy, sensitivity, specificity, and area under the receiver operating characteristic curve (AUC). The GCN model was implemented on Pytorch Geometric Extension library of Pytorch 1.7 in Python 3.7 environment (NVIDIA GeForce RTX 2060 with 8 GB GPU memory). The code in our study can be found at https://github.com/alien18/GCN_SCZ_Classification.

### Identifying the Most Salient Regions Contributing to Classification

We used class activation mapping (CAM) to identify the most salient regions contributing to GCN classification. CAM was originally developed for traditional CNNs to localize the discriminative image area by providing information about regional attention of the CNN model when predicting a particular class.^35^ The novel introduction of CAM into graph-based models enables the localization of discriminative nodes in irregular graph structures beyond regular 2D/3D images.^33^ We estimated the activation value of each node via CAM when predicting patients with schizophrenia (since our task is a binary classification, our findings would have been identical if we had estimated the activation value of each node via CAM when predicting controls). Detailed estimation of CAM can be found in Supplementary Information. Average activation values across subjects were calculated, and the top 10 nodes exhibiting the highest activation values were reported.

### Parcellation Validation

Since no consensus has been reached on the optimal brain parcellation for establishing brain networks, we chose another atlas to assess whether the classification performance of GCN was stable. Considering that the AAL atlas parcellates the brain anatomically, we additionally examined the GCN performance and salient regions using an alternative functional atlas containing 160 regions of interests proposed by Dosenbach et al.^40^ Moreover, the number of parcels in Dosenbach atlas is twice as much compared to AAL atlas, enabling us to validate the performance of GCN across different parcellation resolutions.

### Support Vector Machine

To validate the superiority of the GCN model over brain connectivity, we compared the model performance, salient regions, and clinical correlation of GCN with that of SVM (linear kernel), a traditional machine-learning algorithm commonly used in neuroimaging studies of brain disorders.^41,42^ The upper triangle of the functional connectivity matrix was used as input features. During the training stage, alternative 10-fold nested cross-validation was performed to find the optimal hyperparameters *C* from [10^−3^, 10^−2^, 10^−1^, 1, 10^1^, 10^2^, 10^3^] via grid search. Once the optimal hyperparameter for each fold was determined, SVM was trained again with the whole training set and evaluated on the testing set. To identify regions contributing most to SVM classification, we collected the weights of each functional connectivity value from the trained model. The weight of each region was calculated as the mean value of the weights for its functional connectivity with the other 89 regions across cross-validation. The top 10 regions with the highest weights were reported.

Compared to linear SVM, the nonlinearity in GCN may also account for the difference in model performance. To exclude the influence of nonlinearity in GCN, we further compared the performance of nonlinear SVM with that of GCN (for detail, see Supplementary Information).

### Network Topological Analysis of the Salient Regions

Three nodal topological centralities of the top 10 salient regions, including degree, efficiency, and betweenness, were estimated using the GRETNA toolbox (http://www.nitrc.org/projects/gretna/).^43^ The definition and calculation of nodal topological centralities are described in Supplementary Information. Nonparametric permutation test was performed to examine the significant differences in nodal centralities of salient regions between patients with schizophrenia and controls. The statistical tests were separately performed for top 10 salient regions identified via GCN and SVM. Nodal centralities with FDR corrected *P* value <.05 were reported.

To investigate the relationship between clinical measures and functional network topological profiles of the identified salient regions contributing to classification, we performed a Pearson correlation between positive/negative symptom severity and nodal network centralities of the top 10 salient regions. The correlation analysis was performed for both top 10 salient regions identified by GCN and SVM. As different clinical scales, including the Scale for the Assessment of Positive Symptoms (SAPS), Scale for the Assessment of Negative Symptoms (SANS), and Positive and Negative Syndrome Scale (PANSS), were used in multisite dataset, we converted the raw scores to the Percent of Maximum Possible scores for standardization.^44,45^ FDR correction was applied to address the multiple correlations. Significant correlations with *P* < .05 (corrected for the number of multiple comparisons) were reported.

## Results

### ComBat Harmonization Effects

Prior to ComBat harmonization, 3433 of 4005 functional connectivities (85.7%) exhibited significant cross-site differences in the control group, and the schizophrenia group showed 2844 functional connectivities (71.0%) with significant cross-site effects (FDR corrected *P* < .05). After applying ComBat harmonization, no functional connectivity (0%) in the control group showed significant differences across datasets, and significant sites effects remained in only 111 of 4005 connectivities (2.8%) in the schizophrenia group. Regarding the classification under LOSO cross-validation which can maximize the bias of cross-site effects on model performance, GCN and SVM achieved balanced accuracies of 61.0% and 62.3% prior to ComBat harmonization, respectively. Following the ComBat harmonization, balanced accuracies of 79.1% and 73.4% were observed, with an increase of 18% and 11% for GCN and SVM, respectively.

### Classification Performance

Classification of samples in each single site ranged from 65.7% to 79.2% for GCN and from 67.2% to 75.6% for SVM. Under the 10-fold stratified cross-validation, the performance of GCN achieved an average balanced accuracy of 85.8% (95% CI: 84.9%–86.7%) and AUC value of 0.926 (95% CI: 0.919–0.933). Compared with GCN, a relatively poor performance of SVM was observed, with an average balanced accuracy of 80.9% (95% CI: 79.9%–81.9%) and AUC value of 0.897 (95% CI: 0.889–0.905). When using the LOSO cross-validation scheme, we observed that the balanced accuracy of GCN model was 79.1% (95% CI: 78.0%–80.2%) and AUC value was 0.790 (95% CI: 0.779–0.801), while the SVM classifier only had a balanced accuracy of 73.4% (95% CI: 72.2%–74.6%) and AUC value of 0.753 (95% CI: 0.742–0.764) (table 2 and Supplementary figure S1).

**Table 2.** Performance on classification between individuals with schizophrenia and controls

	GCN			SVM		
Model performance	BAC (%)	SEN (%)	SPE (%)	BAC (%)	SEN (%)	SPE (%)
Dataset 1	68.8	74.5	63.1	73.4	80.1	66.8
Dataset 2	-	-	-	-	-	-
Dataset 3	79.2	78.2	80.2	67.2	63.0	71.4
Dataset 4	79.0	74.3	83.7	75.6	55.8	95.4
Dataset 5	72.3	73.7	71.0	73.8	70.0	77.7
Dataset 6	65.7	52.7	78.6	72.5	53.3	91.7
LOSO (Before ComBat)	61.0	60.4	61.6	62.3	56.4	68.2
LOSO (After ComBat)	79.1	85.0	73.2	73.4	60.6	86.2
10-fold	85.8	74.0	97.6	80.9	69.9	91.9

*Abbreviations:* GCN, graph convolutional network; SVM, support vector machine; BAC, balanced accuracy; SEN, sensitivity; SPE, specificity; LOSO, leave-one-site-out.

When parcellating the brain according to another atlas with 160 regions of interests, the classification performance for both GCN (balanced accuracy: 83.7%) and SVM (balanced accuracy: 75.3%) was stable. This result confirmed that our results are robust to other brain functional parcellation strategies.

### The Most Salient Regions Contributing to Classification

**Fig. 2.**
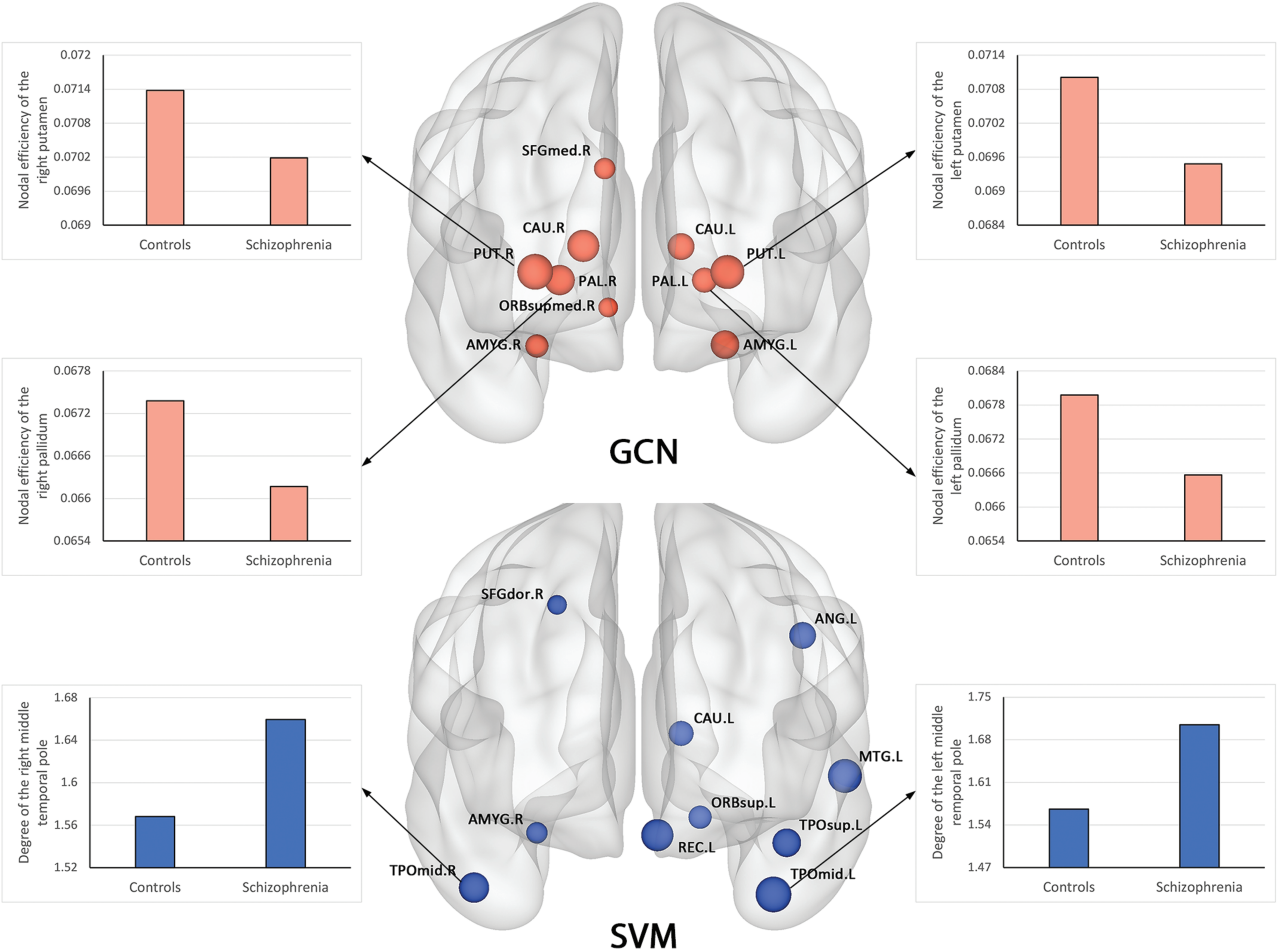
Top 10 salient regions contributing to GCN and SVM classification. The size of each region indicates the magnitude of contribution. Bar plots are used to illustrate statistically significant differences in topological characteristics between patients with schizophrenia and controls. *Note*: AMYG, amygdale; ANG, angular gyrus; CAU, caudate; GCN, graph convolutional network; MTG, middle temporal gyrus; ORBsup, orbitofrontal gyrus, superior part; ORBsupmed, orbitofrontal gyrus, superior medial part; PAL, pallidum; PUT, putamen; REC, rectus; SFGdor, superior frontal gyrus, dorsal part; SFGmed, superior frontal gyrus, medial part; SVM, support vector machine; TPOmid, temporal pole, middle part; TPOsup, temporal pole, superior part.

The 10 most salient regions contributing to GCN classification were mainly located in subcortical and frontal structures, including the striatum, amygdale, and medial superior frontal gyrus. Regarding the SVM model, the top 10 regions contributing to classification were extensive cortical areas, including temporal gyrus, angular gyrus, dorsolateral prefrontal cortex, and orbitofrontal cortex (Supplementary table S1 and figure 2). When using Dosenbach atlas for brain parcellation, consistent pattern of top 10 salient regions was observed for both GCN and SVM (Supplementary table S2).

### Topological Characteristics of the Most Salient Regions

Using GCN, we found that patients with schizophrenia exhibited significantly decreased nodal efficiency in the bilateral putamen and pallidum compared with controls. No significant between-group differences in the most salient regions were observed for degree and betweenness. Using SVM, the degree of the bilateral middle temporal pole was significantly higher in patients with schizophrenia compared with controls. Differences in efficiency and betweenness of the other most salient regions failed to survive multiple comparison correction (Supplementary table S1 and figure 2).

### Statistical Analysis

**Fig. 3.**
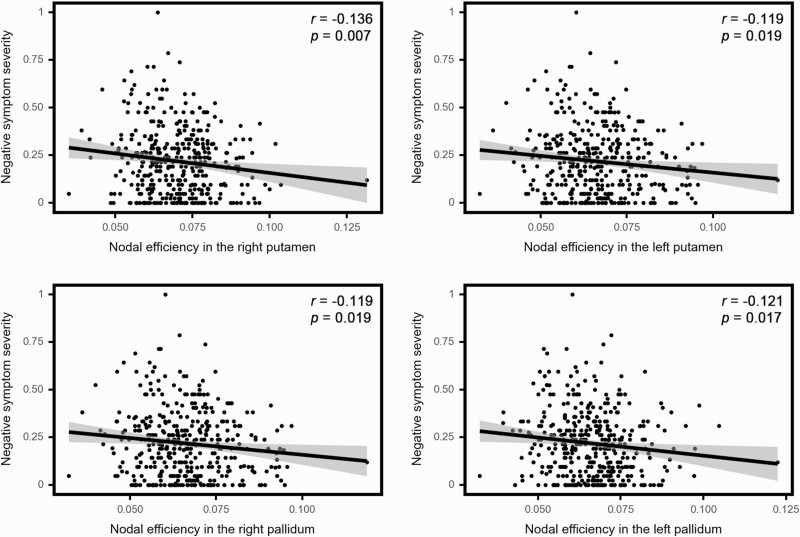
Correlation between nodal efficiency in the bilateral putamen and pallidum with severity of negative symptoms in individuals with schizophrenia.

In the correlation analysis of the most salient regions derived from GCN, we found that nodal efficiency of bilateral putamen and pallidum were significantly associated with negative symptom scores (figure 3). No significant relationship that survived FDR correction was observed between clinical scores and other nodal metrics of the top 10 salient regions derived from GCN. Among the salient regions identified by SVM, no significant correlation between nodal centralities and positive/negative symptom scores were found (Supplementary table S1).

## Discussion

In the current study, we classified individuals with schizophrenia from controls across multiple sites with an accuracy of 85.8% using a GCN model. Compared with a widely used traditional machine-learning method (ie, linear SVM), GCN yielded an improved balanced accuracy of approximately 5%, suggesting the potential of graph-based deep learning approaches for discrimination based on functional connectivity features. This promising result may derive from the 2 distinct aspects of our investigation, which will be discussed in turn.

First, graph-based learning approaches may better fit the brain network topological structure by considering neighborhood relationships within a network beyond independent connectivities, ensuring the integrity of information during the extraction of hidden representations. Previous studies have indicated that connectomes, constructed from neuroimaging data, can be a powerful tool for characterizing brain disorders and drug treatment effects at the level of the individual patient.^8,9,46^ Networks may play an essential role in the “dysconnectivity” model underlying the pathophysiology of schizophrenia. Deep learning methods may be capable of learning reliable connectome patterns and help understand the pathophysiology and achieve accurate identification of schizophrenia across multiple independent imaging sites. For example, Zeng et al combined deep discriminant autoencoder network and brain connectome features, obtaining an accuracy within the range 81%–85% based on a multisite schizophrenia dataset that partial overlaps with our dataset.^47^ Both their study and our study achieved a higher classification performance than other multisite classification efforts, suggesting that the application of powerful deep learning methods to measure of brain connectivity is a promising approach for the diagnosis of schizophrenia.

Second, previous studies have suggested that the deep learning architectures require a bigger sample size than “shallow” machine-learning models such as SVM.^23^ In our investigation of single-site datasets, we noted that GCN performed worse than SVM in some cases. This may be due to the sample size requirements related to model complexity in deep learning. Despite the advantage of graph representation in GCN, higher risk of overfitting and insufficient generalizability can still cause a significant impairment on the performance of GCN in the context of small datasets. For this reason, we combined datasets from multiple sites to increase the training sample size. However, one of the key challenges in neuroimaging studies of brain disorders is the poor generalizability of the findings across independent datasets, possibly due to different recruitment criteria, scanners, and scanning parameters. For example, our previous investigation, using LOSO cross-validation without removing site-related differences, has indicated high within-site performance but poor generalizability across different sites.^48^ After using ComBat harmonization, our multiple site classification based on LOSO cross-validation shows promising results. This performance confirms that multisite neuroimaging studies can benefit from the use of feature harmonization methods for removing site-related differences, especially in the context of deep learning architectures that work better on large sample size.

Within the GCN model, we found that the striatal areas (ie, putamen, pallidum, and caudate) and amygdala were among the areas providing the greatest contribution. The mesolimbic hypothesis, positing that aberrant functioning of midbrain dopamine projections to limbic regions causes psychotic symptoms,^49,50^ has been an influential model of schizophrenia for several decades. Dysregulated dopaminergic modulation of striatal function is still fundamental to many models that seek to explain the mechanisms underlying psychotic symptoms.^51–53^ Among the regions providing the greatest contribution, decreased nodal efficiency in the bilateral putamen and pallidum were found in patients relative to controls. This is consistent with our second hypothesis that GCN would provide distinct saliency maps of brain regions, reflecting the ability of this technique to capture network-level topological information. Moreover, the bilateral putamen and pallidum’s nodal efficiency was significantly correlated with negative symptoms, consistent with previous studies suggesting that the putamen may be a key structure for the neurobiological underpinning of delusions^54^ and is a possible predictor of clinical course and risk-stratifier in people at clinical high risk for psychosis.^55^ Interestingly, increased volume of putamen has been found to be a transdiagnostic neuroanatomical feature of psychiatric illness^56,57^ and to be positively correlated with severity of symptoms,^56^ while larger-than-normal volumes in the pallidum has also been reported in patients with schizophrenia.^58^ The amygdala, part of the limbic system, provided the greatest contribution within both GCN and SVM models. This region is responsible for processing emotional aspects of face processing,^59,60^ and its dysregulation has been widely implicated in the pathophysiology of schizophrenia, with several studies reporting abnormal functional connectivity in patients relative to healthy controls.^61^ Our investigation extends previous findings based on group-level statistics^62^ by suggesting that striatal and amygdala functional dysconnectivity is key for differentiating patients and controls at individual level.

In contrast, within the SVM model, the brain regions providing the greatest contribution to single-subject classification were mainly located in the temporal cortex. Volumetric abnormalities in the temporal lobe have been reported to be related to clinical presentation, especially negative symptoms.^63,64^ However, no significant correlation was found between SVM-derived salient regions and either positive or negative symptoms scores. Taken collectively, these results are consistent with the notion that the GCN model can be used to detect spatially segregated salient regions in non-Euclidean space^33,35^ and that this approach can capture clinically relevant neuropathological alterations with greater sensitivity than traditional machine-learning models.

The present study has several limitations. First, the results described in this article require replication before potential clinical translation can be considered, especially if one was to generalize our GCN model to unseen sites with highly imbalanced class problems (such as 1:1000). Second, antipsychotic medication may lead to changes in brain function,^65^ which may have contributed to classification. However, our results were statistically consistent across the 5 datasets, including dataset 1 in which all patients were medication-naive; this suggests that our findings are unlikely to be explained by the effects of antipsychotic medication. Third, other GNN models may have the potential to outperform GCN. Future work should focus on the comparison among GNN models with the aim of determining the optimal GNN model for schizophrenia diagnosis. Fourth, saliency maps can suffer from issues such as gradient saturation which may affect robustness; the application of other post hoc explainability methods, like GNNexplainer^66^ or GraphLIME,^67^ may yield more robust explanations in future studies. Fifth, since various graph modeling methods have been used in GCN applications and no consensus has been reached so far, alternative approaches for establishing the optimal graph structure should be investigated in the future. Finally, the application of GCN to neuroimaging data required specialized technical expertise as well as high computational resources, which are not common across clinical sites. Therefore, to improve the likelihood of successful clinical translation in the future, one would need to bridge the current gap between “models” and “tools.” ^68^

In conclusion, the present study demonstrates that GCN allows the classification of schizophrenia at the individual level with significant accuracy, indicating a promising direction for detecting individual patients with schizophrenia. Moreover, striatal areas and amygdala were found among the most salient brain regions in our GCN model, with a number of significant associations with negative symptoms. These findings support the notion that the topology of striatal areas including putamen and pallidum may represent a core neural deficit of negative symptomatology in schizophrenia.

## Supplementary Material

sbac047_suppl_Supplementary_Material
