# Supplementary material for: Graph Convolutional Networks Reveal Network-Level Functional Dysconnectivity in Schizophrenia
Source: Schizophr Bull. 2022 May 15;48(4):881–92. doi: 10.1093/schbul/sbac047 (PMC9212102; doi:10.1093/schbul/sbac047)
Supplement: sbac047_suppl_Supplementary_Material [file sbac047_suppl_supplementary_material.docx]

**Supplemental Methods**

***Participants***

Datasets 1 (301 patients and 217 controls) and 2 (340 controls) were acquired at the West China Hospital of Sichuan University, Chengdu (China) ^1^. An initial diagnosis of schizophrenia and duration of illness were determined by consensus between two experienced psychiatrists, using the Structured Clinical Interview for DSM-IV (SCID)-Patient Version. In addition, diagnosis of schizophrenia was confirmed for all the patients at 1-year follow-up. Exclusion criteria were the existence of a neurological disorder or other psychiatric disorders, alcohol or drug abuse, pregnancy, and any chronic physical illness such as a brain tumor, hepatitis, or epilepsy, as assessed by clinical evaluations and medical records. The study was approved by the ethics committee of West China Hospital, and written informed consent was obtained from all participants.

Dataset 3 (49 patients and 63 controls) was acquired at Maastricht University (The Netherlands) ^2^. Patients were recruited through clinicians working in selected representative geographic areas in the Netherlands and Belgium. Diagnosis of schizophrenia was based on DSM-IV criteria, assessed with the Comprehensive Assessment of Symptoms and History (CASH) interview ^3^. Exclusion criteria included confirmed or suspected pregnancy, any history of neurological disorders, a history of intellectual disability and/or a history of substance abuse/dependence within the last 12 months. The ethics committee of Maastricht University approved the study, and all the participants gave written informed consent in accordance with the committee's guidelines and the Declaration of Helsinki.

Dataset 4 (32 patients and 83 controls) was acquired at the Trinity College Institute of Neuroscience in Dublin (Ireland) as part of a Science Foundation Ireland-funded neuroimaging genetics study (“A structural and functional MRI investigation of genetics, cognition and emotion in schizophrenia”) ^2^. Patients with confirmed DSM-IV diagnosis of schizophrenia were recruited through local clinical services. Exclusion criteria included confirmed or suspected pregnancy, any history of neurological disorders or intellectual disability and substance misuse in the preceding 3 months. Participants provided written informed consent in accordance with local ethics committee guidelines.

Dataset 5 (68 patients and 72 controls) was obtained from the Neuroimaging Informatics Tools and Resources Clearinghouse (NITRC) website and was provided by the Centers of Biomedical Research Excellence (COBRE) (<http://fcon_1000.projects.nitrc.org/indi/retro/cobre.html>) ^4^. In this dataset, a diagnosis of schizophrenia was made using the Structured Clinical Interview for DSM-IV Disorders (SCID; Diagnostic and Statistical Manual of Mental Disorders, DSM-IV). Exclusion criteria included confirmed or suspected pregnancy, any history of neurological disorders and a history of intellectual disability. Written informed consent was obtained from participants according to institutional guidelines at the University of New Mexico.

Dataset 6 (56 patients and 132 controls), acquired as part of the UCLA Consortium for Neuropsychiatric Phenomics LA5c Study ^5^, was obtained from the OpenfMRI database (accession number: ds000030, <https://openneuro.org/datasets/ds000030/versions/1.0.0>) ^6^. All patients underwent a semi-structured assessment with the Structured Clinical Interview for DSM-IV disorders. Exclusion criteria included left-handedness, pregnancy, history of head injury with loss of consciousness or cognitive sequelae, or other contraindications to scanning. After receiving a verbal explanation of the study, participants gave written informed consent following procedures approved by the Institutional Review Boards at UCLA and the Los Angeles County Department of Mental Health.

***Data acquisition***

Each dataset comprised of resting state functional Magnetic Resonance Imaging (fMRI) images were acquired. Dataset 1 was acquired using a 3T GE scanner (EXCITE; General Electric, Milwaukee, Wisconsin). The sequence parameters were as follows: repetition time/ echo time (TR/TE) = 2000/30 ms; flip angle = 90°; 30 axial slices per volume; voxel size = 3.75 × 3.5 × 5mm^3^; number of volumes = 200. Dataset 2 was acquired using a 3T Siemens scanner. The sequence parameters were as follows: repetition time/ echo time (TR/TE) = 2000/30 ms; flip angle = 90°; 30 axial slices per volume; voxel size = 3.75 × 3.5 × 5mm^3^; number of volumes = 200. Dataset 3 was acquired using a 3T Siemens Magnetom Allegra head scanner. The sequence parameters were as follows: repetition time/echo time (TR/TE) = 1500/30 ms; flip angle = 90°; 27 axial slices per volume; voxel size = 3.5 × 3.5 × 5.2 mm^3^; number of volumes = 200. Dataset 4 was acquired using a 3T Philips Intera Achieva scanner. The sequence parameters were as follows: repetition time/echo time (TR/TE) = 2000/30 ms; flip angle = 90°; 35 axial slices per volume; voxel size = 3.5 × 3.5 × 3.5 mm^3^; number of volumes = 180. Dataset 5 was acquired using a 3T Siemens scanner. The sequence parameters were as follows: repetition time/echo time (TR/TE) = 2000/30 ms; flip angle = 90°; 33 axial slices per volume; voxel size = 3.75 × 3.75 × 4.55 mm^3^; number of volumes = 150. Dataset 6 was acquired using a 3T Siemens scanner. The sequence parameters were as follows: repetition time/echo time (TR/TE) = 2000/30 ms; flip angle = 90°; 34 axial slices per volume; voxel size = 3 × 3 × 4mm^3^; number of volumes = 152.

***Image Preprocessing***

A unified functional image preprocessing pipeline was used for each dataset. Specifically, the first 10 time points were discarded to minimize the impact of the instability in the initial MRI signal. The remaining images were corrected for intravolume acquisition time delay. To minimize the potential impact of head motion artifacts—a recognized challenge in resting-state fMRI analyses ^7, 8^, we applied Friston 24-parameter correction ^9^ and the “head motion scrubbing” method proposed by Power and colleagues ^10^ to ensure that motion artifacts were not contributing to the group differences we observed. For each participant, volumes with framewise displacement (FD) greater than 0.5 mm were identified and excluded. After these corrections, the images were spatially normalized to a 3 × 3 × 3mm^3^ Montreal Neurological Institute (MNI) 152 template and then linearly detrended and temporally bandpass filtered (0.01–0.08 Hz) to remove low-frequency drift and high-frequency physiological noise. Finally, the global signal ^11^, the white matter signal, the cerebrospinal fluid signal and the motion parameters were regressed out. None of the subjects included in the present investigation showed excessive head motion during scanning (defined as translational movement >1.5 mm and/or rotation >1.5°).

***ComBat harmonization***

ComBat was originally developed to adjust batch effects in genomic studies ^12^. Since then, it has been validated as an effective method for reducing site-related variability in multi-site structural, functional and diffusion MRI data ^13-15^. ComBat is essentially a multivariate linear mixed effects regression model with additive and multiplicative terms for site effect. Empirical Bayes was used during the modelling process to improve the estimation of biological and site effect parameters. By removing site-effect variance and preserving biological variance of interest, ComBat provides a balanced way simultaneously to correct measurements from multi-site data and avoid overcorrection on important biological variance. Herein, we performed ComBat harmonization over functional connectivity values stored in the upper triangles of each connectivity matrix, and the case-control difference was included as a covariate of interest to be protected. To avoid potential information leakage, we independently performed ComBat harmonization in training set and applied the estimated model to test set data. In addition, we also quantified the effects of ComBat harmonization to see how it works on our dataset.

To quantify the magnitude of differences in functional connectivity across datasets, we used Kruskal–Wallis tests to assess the site effects for each functional connectivity value. False discovery rate (FDR) correction was used to adjust the *p* values derived from multiple comparisons. Functional connectivity with a corrected *p* value < 0.05 would be regarded as the existence of a significant site effect. We separately assessed the site effects in the schizophrenia and control groups. The numbers and percentages of significantly different connectivities across sites prior to and following ComBat harmonization were reported. In addition, we further compared the classification performance prior to and following ComBat harmonization.

***Spectral graph convolution***

The core process of our GCN model is the spectral graph convolution, which can implement the convolution operation on irregular graph data. Spectral graph convolution firstly requires the typical graph being transformed into spectral domain. To this end, eigen-decomposition is applied on normalized Laplacian of a given graph $G$ and followed by graph Fourier transform (GFT) to convert spatial graph convolution into spectral multiplication ^16^. Specifically, the normalized Laplacian $L$ is given by $L=I_{a}-D^{-1/2}WD^{-1/2}$, where $I_{a}$ is the identity matrix and $D$ is the degree matrix. Given the semidefinite property of Laplacian, it can be decomposed as $L=U\wedge U^{T}$, where $U$ includes the eigenvectors and $\wedge$ is the diagonal matrix of eigenvalues. Assuming the node feature $x$ and graph convolutional filter $g_{\theta}$, spatial graph convolutional can be expressed as spectral multiplication:

$$g_{\theta}*x= g_{\theta}\left( L \right)x=g_{\theta}\left( U\wedge U^{T} \right)x=Ug_{\theta}\left( \wedge\right)U^{T}x$$

To reduce the computation expense and strictly localize the filters, polynomial parametrization on normalized Laplacian is applied. Using a polynomial of order $K$ exactly defines $K$-localized filters which limit the number of neighbors considered during the convolution operation. Defferrard et al. recommended using $K$-order Chebyshev polynomials $\sum_{k=0}^{K} \beta_{k}T_{k}(\tilde{\wedge})$instead of the filter $g_{\theta}\left( \wedge\right)$, where $T_{k}$ denotes the polynomials $\beta_{k}$ denotes the polynomial coefficient ^17^. Herein, scaled diagonal matrix of eigenvalues $\tilde{\wedge}= \frac{2\wedge}{\lambda_{max}}-I_{a}$ is used to fit the input range of Chebyshev polynomials (i.e., [-1, 1]), where $\lambda_{max}$ is the largest eigenvalue of Laplacian. Chebyshev polynomials are recursively defined as $T_{k}\left( L_{i} \right)=2L_{i}T_{k-1}\left( L_{i} \right)-T_{k-2}$ with $T_{0}\left( L_{i} \right)=1$ and $T_{1}\left( L_{i} \right)=L_{i}$. Therefore, a $K$-localized graph convolution can be further written as:

$$g_{\theta}*x=U\sum_{k=0}^{K} \beta_{k}T_{k}\left( \tilde{\wedge} \right)U^{T}x=\sum_{k=0}^{K} \beta_{k}T_{k}\left( U\tilde{\wedge}U^{T} \right)x=\sum_{k=0}^{K} \beta_{k}T_{k}\left( \tilde{L} \right)x$$

, with $\tilde{L}=\frac{2L}{\lambda_{max}}-I_{a}$. Finally, the output of the $l^{th}$ graph convolutional layer for a sample $i$ is given by:

$$y_{i}^{l}=\sum_{j=1}^{F_{in}} g_{\theta_{j}^{l}}(L)x_{i,j}^{l}$$

Where $F_{in}$ and $F_{out}$ denotes the number of input and output filters, respectively, $x_{i,j}^{l}$ is the input feature map $j$ for sample $i$ at layer $l$ and $\theta_{j}^{l}\in\mathbb{R}^{K}$ denotes the trainable coefficients of $K$-order Chebyshev polynomials. Hence, the number of all trainable parameters for a graph convolutional layer is $F_{in}\times F_{out}\times K$.

***GCN hyperparameters***

After building a graph for the individual feature matrix, the GCN model was trained to make a categorical prediction about each subject (i.e., patient vs control). The core process of our GCN model is the spectral graph convolution layer, which can be used to implement the convolution operation on irregular graph data. To determine the hyperparameters of GCN, we randomly extracted 1/10 data from the training set for validation in each fold. Different settings of hyperparameters were manually examined, and the hyperparameter set with the best validation accuracy was used. After model setup, the final model was refit using the whole training set and subsequently evaluated on the testing set. Finally, the overall architecture of our GCN model contains 3 graph convolutional layers (number of channels: [64, 64, 128]) followed by a global average pooling layer and a fully connected layer. Hidden layers are activated by the Rectified Linear Unit (ReLU) function to introduce non-linearity into the model, and the output layer is activated by the Softmax function to encode the output value into predictive probability for each class. Cross entropy loss function and Adam optimization algorithm are used to quantify the loss and update the model parameters, respectively. To reduce the overfitting risk, the dropout strategy is used at the 2nd and 3rd layers with a rate of 0.5, and a L_2 regularization term of 5×10-4 is included in the loss function. The initial learning rate is set as 0.001, and the order of Chebyshev polynomials is 6. The model training is conducted based on a mini-batch of 64 samples for 50 epochs.

***Sensitivity analysis of k values in KNN***

To determine the optimal k value in KNN algorithms, we examined the GCN performance with dynamic k values from [5, 10 ,15, 20]. Ten-fold pooled stratified cross-validation was applied to split training and testing dataset. The GCN hyperparameters were consistent across different k values. The balanced accuracy of GCN model under different k values were recorded and reported. We found that GCN achieved comparable performance when k = 5 or 10, with balanced accuracies of 85.0% and 85.8%, respectively. When increasing k value up to 15 or 20, we obtained poor performance with balanced accuracies of 81.1% and 77.6% (Figure S2).

***Class activation mapping***

Assuming the activation value $y_{i}\left( v \right)$ in the last graph convolutional layer at node $v$ for the feature map $i$, the following global average pooling operation can be defined as $Y_{i}=(1/n)\sum y_{i}\left( v \right)$, where $n$ denote the number of nodes. The output value of fully connected layer for class $c$ is calculated as $\sum w_{i}^{c}Y_{i}$. We extract the weight $w_{i}^{c}$ of $Y_{i}$ for class $c$ and the class activation value of each node $v$ for a given class $c$ was calculated as $A_{c}\left( v \right)=\sum w_{i}^{c}y_{i}(v)$. Since the classification task is binary, CAM for either patients or controls can reflect the regional contribution to classification. We thus summed up and averaged the activation values of each region across all the subjects.

***Non-linear SVM***

Considering that the non-linearity in GCN may also account for better model performance over linear SVM, we examined the performance of a non-linear SVM classifier (i.e., SVM with radial basis function (RBF) kernel) for comparison. Consistent with linear SVM, the functional connectivity values between each pair of regions were used as features. Ten-fold stratified cross-validation and LOSO cross-validation were separately applied to examine the model performance. During the training stage, a nested 10-fold cross-validation was further applied for grid search to optimize the hyperparameters C from [10^-3^, 10^-2^, 10^-1^, 1, 10^1^, 10^2^, 10^3^] and Gamma from [10^-2^, 10^-1^, 1, 10^1^, 10^2^]. Once the optimal hyperparameter for each fold was determined, the RBF-SVM was trained again using the whole training set and evaluated on the testing set. The model performance was assessed in terms of balanced accuracy, sensitivity, specificity and AUC.

For the 10-fold stratified cross-validation, RBF-SVM achieved balanced accuracy of 77.0%, sensitivity of 66.7%, specificity of 87.0% and AUC value of 0.85. Under LOSO cross-validation, RBF-SVM achieved balanced accuracy of 71.6%, sensitivity of 61.6%, specificity of 81.6% and AUC value of 0.80. Therefore, the performance of non-linear SVM was worse than that of linear SVM, indicating that the non-linearity is unlikely to account for the better performance of GCN.

***Network topological analysis***

Three nodal topological centralities of the top 10 salient regions, including degree, efficiency and betweenness, were estimated using the GRETNA toolbox (<http://www.nitrc.org/projects/gretna/>) ^18^. Degree is the simplest metric to delineate the importance of a node in a graph, measuring the summed connections of the index node with all the other nodes. Betweenness is a measure of the influence of a given node on information flow in the graph, and is calculated as the fraction of all shortest paths in the graph that pass through a given node. Efficiency is defined as the ability to support information flow of a given node, and is calculated as inversely proportional to the mean shortest distance between index node and all the other nodes.

A wide range of sparsity thresholds were applied to all harmonized network matrices to provide accurate estimation of nodal topological properties. The sparsity was determined as the percentage of edges with highest connectivity values, and was chosen to ensure that networks were estimable for the small-worldness scalar and the small-world index (σ) was larger than 1.0 ^19^. The range of our sparsity thresholds was set from 0.1 to 0.34 with an interval of 0.01. For each metric, the AUC was calculated under the sparsity range, which can provide a summarized scalar for the topological characterization of brain networks independent of a single threshold selection. The AUC metric has proven to be sensitive in the detection of topological alterations of brain networks.

**Figure S1.** The receiver operating characteristic curves for GCN and SVM classification.


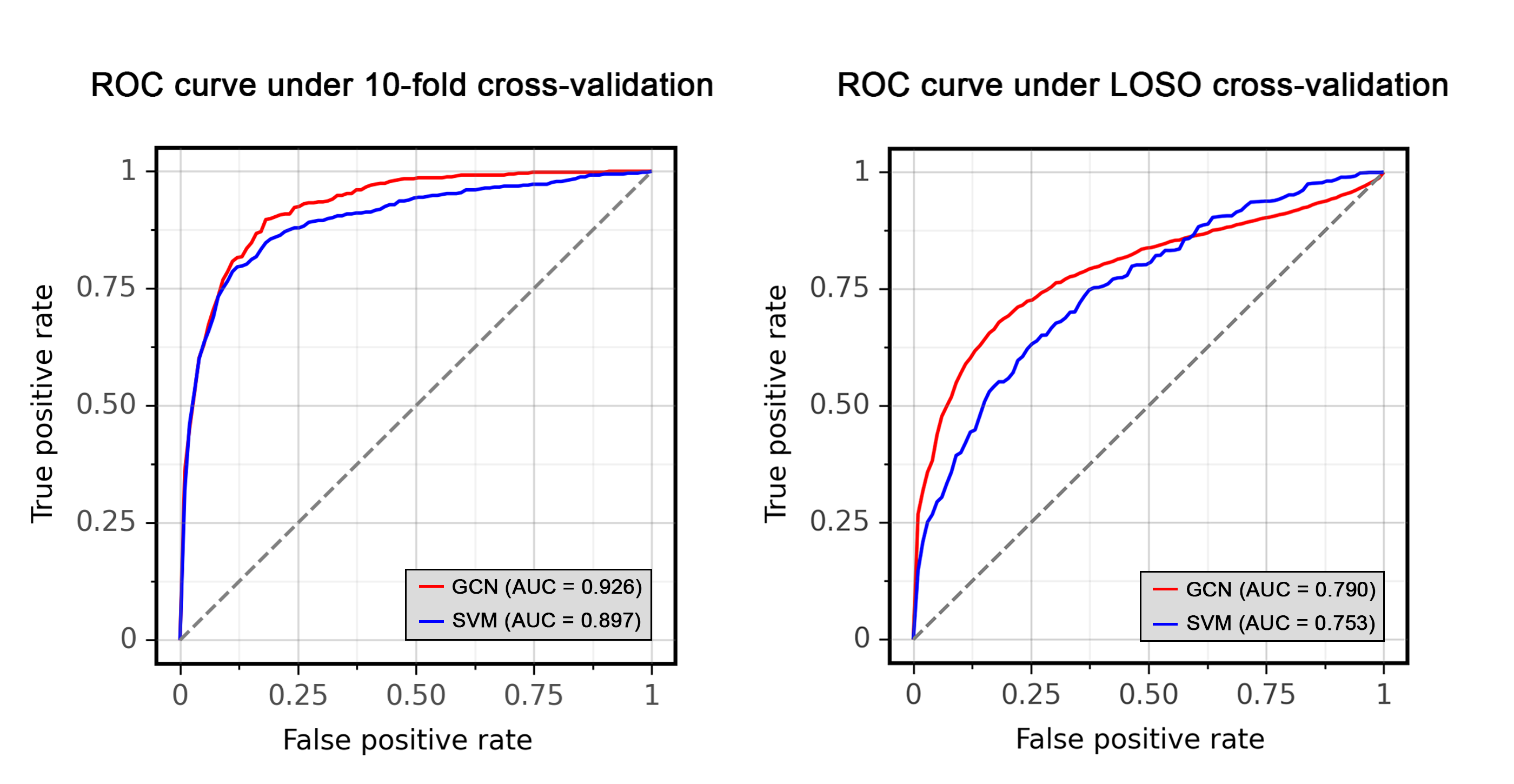
*Abbreviations*: ROC, receiver operating characteristic; GCN, graph convolutional network; SVM, support vector machine.


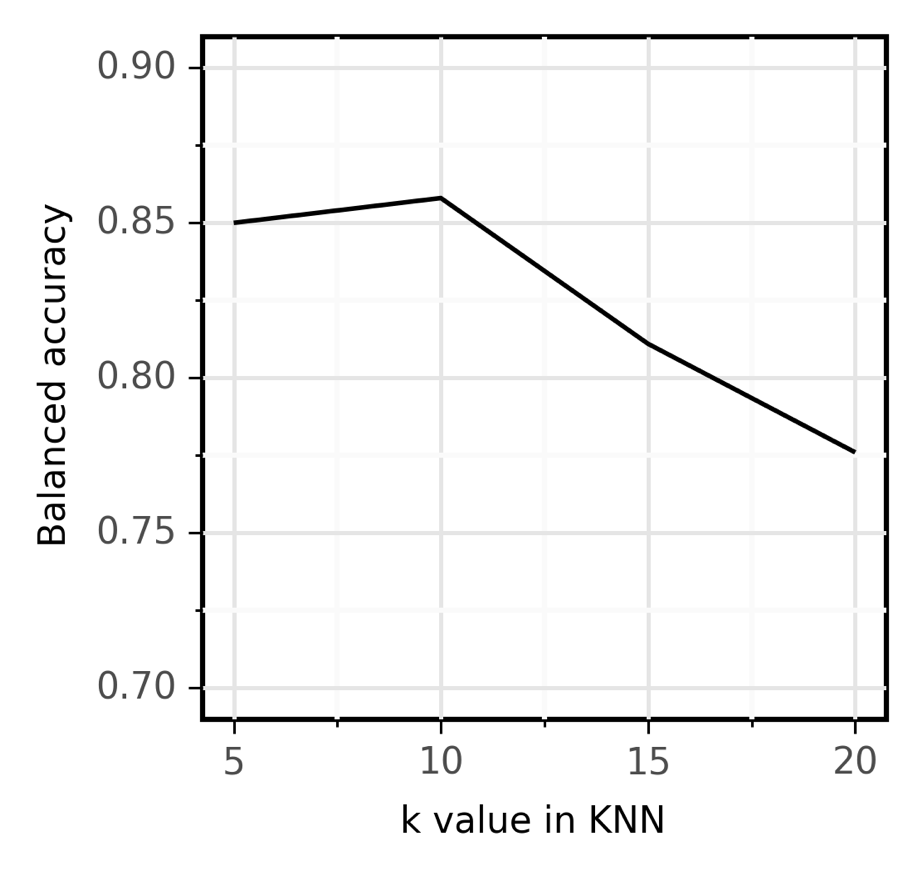
**Figure S2** GCN performance under different k values in KNN.

| **Table S1** Top 10 salient regions contributing to the GCN and SVM and results of topological analysis. | | | | | | | | | | | |
| --- | --- | --- | --- | --- | --- | --- | --- | --- | --- | --- | --- |
| Rank | Brain Region | *P* value of topological difference | | |  | *P* value of correlation between topological metrics and symptoms | | | | | |
|  |  | Degree | Efficiency | Betweenness |  | Degree | | Efficiency | | Betweenness | |
|  |  |  |  |  |  | Positive symptom | Negative symptom | Positive symptom | Negative symptom | Positive symptom | Negative symptom |
| GCN |  |  |  |  |  |  |  |  |  |  |  |
| 1 | R putamen | 0.071 | **0.017** | 0.286 |  | 0.404 | 0.018 | 0.173 | **0.007** | 0.741 | 0.716 |
| 2 | L putamen | 0.017 | **0.004** | 0.107 |  | 0.666 | 0.060 | 0.331 | **0.019** | 0.689 | 0.645 |
| 3 | R Caudate | 0.481 | 0.431 | 0.419 |  | 0.166 | 0.634 | 0.796 | 0.594 | 0.185 | 0.691 |
| 4 | R Pallidum | 0.147 | **0.019** | 0.269 |  | 0.666 | 0.060 | 0.331 | **0.019** | 0.689 | 0.645 |
| 5 | L Amygdala | 0.147 | 0.130 | 0.421 |  | 0.503 | 0.198 | 0.095 | 0.069 | 0.806 | 0.645 |
| 6 | L Caudate | 0.250 | 0.286 | 0.028 |  | 0.128 | 0.382 | 0.614 | 0.752 | 0.134 | 0.084 |
| 7 | L Pallidum | 0.043 | **0.010** | 0.444 |  | 0.501 | 0.080 | 0.166 | **0.017** | 0.112 | 0.338 |
| 8 | R Amygdala | 0.500 | 0.265 | 0.061 |  | 0.398 | 0.628 | 0.169 | 0.230 | 0.696 | 0.356 |
| 9 | R SFG, medial | 0.008 | 0.087 | 0.017 |  | 0.665 | 0.177 | 0.903 | 0.071 | 0.827 | 0.410 |
| 10 | R SFG, medial orbital | 0.052 | 0.108 | 0.231 |  | 0.731 | 0.162 | 0.507 | 0.076 | 0.433 | 0.069 |
| SVM |  |  |  |  |  |  |  |  |  |  |  |
| 1 | L Temporal pole: MTG | **0.005** | 0.028 | 0.097 |  | 0.345 | 0.867 | 0.291 | 0.856 | 0.649 | 0.680 |
| 2 | L MTG | 0.203 | 0.322 | 0.275 |  | 0.395 | 0.325 | 0.807 | 0.419 | 0.109 | 0.026 |
| 3 | L Rectus gyrus | 0.097 | 0.149 | 0.333 |  | 0.051 | 0.884 | 0.082 | 0.894 | 0.244 | 0.766 |
| 4 | R Temporal pole: MTG | **0.005** | 0.046 | 0.468 |  | 0.059 | 0.628 | 0.107 | 0.151 | 0.297 | 0.889 |
| 5 | L Temporal pole: STG | 0.429 | 0.254 | 0.201 |  | 0.399 | 0.300 | 0.693 | 0.163 | 0.023 | 0.913 |
| 6 | L Angular gyrus | 0.031 | 0.027 | 0.189 |  | 0.629 | 0.751 | 0.534 | 0.718 | 0.075 | 0.117 |
| 7 | L Caudate | 0.250 | 0.286 | 0.028 |  | 0.128 | 0.382 | 0.614 | 0.752 | 0.134 | 0.084 |
| 8 | L SFG, orbital part | 0.108 | 0.044 | 0.341 |  | 0.880 | 0.749 | 0.528 | 0.339 | 0.196 | 0.123 |
| 9 | R Amygdala | 0.500 | 0.265 | 0.061 |  | 0.398 | 0.628 | 0.169 | 0.230 | 0.696 | 0.356 |
| 10 | R SFG, dorsolateral | 0.067 | 0.133 | 0.060 |  | 0.782 | 0.445 | 0.710 | 0.476 | 0.713 | 0.793 |

*Abbreviations*: GCN, graph convolutional network; SVM, support vector machine; SFG, superior frontal gyrus; MTG, middle temporal gyrus; STG, superior temporal gyrus.

P values shown in bold survived a significance level of FDR corrected p < 0.05.

**Table S2** Top 10 salient regions contributing to the GCN and SVM based on Dosenbach functional atlas.

| Rank | GCN | SVM |
| --- | --- | --- |
| 1 | R putamen | L Temporal pole: MTG |
| 2 | L putamen | L MTG |
| 3 | R Caudate | R Temporal pole: MTG |
| 4 | L putamen | R Temporal pole: MTG |
| 5 | L pallidum | L Rectus gyrus |
| 6 | L Caudate | L Temporal pole: MTG |
| 7 | R SFG, medial orbital | L Temporal pole: MTG |
| 8 | R pallidum | L SFG, orbital part |
| 9 | L Amygdala | L Caudate |
| 10 | R SFG, medial | L SFG, orbital part |

Abbreviations: GCN, graph convolutional network; SVM, support vector machine; SFG, superior frontal gyrus; MTG, middle temporal gyrus.

*Note*: To obtain comparable findings, the label of salient regions in Dosenbach atlas were based on the annotation of AAL atlas. The coordinates of regions in Dosenbach atlas were extracted and projected into the AAL atlas to obtain the annotation. The size of ROI in the Dosenbach atlas is significantly smaller than that in the AAL atlas, thus different ROIs in the former can be projected into the same region in the latter.

**References**

**1.** Sun H, Lui S, Yao L, et al. Two Patterns of White Matter Abnormalities in Medication-Naive Patients With First-Episode Schizophrenia Revealed by Diffusion Tensor Imaging and Cluster Analysis. *JAMA Psychiatry* Jul 2015;72(7):678-686.

**2.** Morgan SE, Seidlitz J, Whitaker KJ, et al. Cortical patterning of abnormal morphometric similarity in psychosis is associated with brain expression of schizophrenia-related genes. *Proc Natl Acad Sci U S A* May 7 2019;116(19):9604-9609.

**3.** Andreasen NC, Flaum M, Arndt S. The Comprehensive Assessment of Symptoms and History (CASH). An instrument for assessing diagnosis and psychopathology. *Arch Gen Psychiatry* Aug 1992;49(8):615-623.

**4.** Aine CJ, Bockholt HJ, Bustillo JR, et al. Multimodal Neuroimaging in Schizophrenia: Description and Dissemination. *Neuroinformatics* Oct 2017;15(4):343-364.

**5.** Gorgolewski KJ, Durnez J, Poldrack RA. Preprocessed Consortium for Neuropsychiatric Phenomics dataset. *F1000Res* 2017;6:1262.

**6.** Poldrack RA, Congdon E, Triplett W, et al. A phenome-wide examination of neural and cognitive function. *Sci Data* Dec 6 2016;3:160110.

**7.** Power JD, Barnes KA, Snyder AZ, Schlaggar BL, Petersen SE. Spurious but systematic correlations in functional connectivity MRI networks arise from subject motion. *NeuroImage* Feb 1 2012;59(3):2142-2154.

**8.** Satterthwaite TD, Elliott MA, Gerraty RT, et al. An improved framework for confound regression and filtering for control of motion artifact in the preprocessing of resting-state functional connectivity data. *NeuroImage* Jan 1 2013;64:240-256.

**9.** Yan CG, Craddock RC, He Y, Milham MP. Addressing head motion dependencies for small-world topologies in functional connectomics. *Frontiers in human neuroscience* 2013;7:910.

**10.** Power JD, Mitra A, Laumann TO, Snyder AZ, Schlaggar BL, Petersen SE. Methods to detect, characterize, and remove motion artifact in resting state fMRI. *NeuroImage* Jan 1 2014;84:320-341.

**11.** Fox MD, Zhang D, Snyder AZ, Raichle ME. The global signal and observed anticorrelated resting state brain networks. *Journal of neurophysiology* Jun 2009;101(6):3270-3283.

**12.** Johnson WE, Li C, Rabinovic A. Adjusting batch effects in microarray expression data using empirical Bayes methods. *Biostatistics (Oxford, England)* Jan 2007;8(1):118-127.

**13.** Yu M, Linn KA, Cook PA, et al. Statistical harmonization corrects site effects in functional connectivity measurements from multi-site fMRI data. *Human brain mapping* Nov 2018;39(11):4213-4227.

**14.** Fortin JP, Cullen N, Sheline YI, et al. Harmonization of cortical thickness measurements across scanners and sites. *NeuroImage* Feb 15 2018;167:104-120.

**15.** Fortin JP, Parker D, Tunç B, et al. Harmonization of multi-site diffusion tensor imaging data. *NeuroImage* Nov 1 2017;161:149-170.

**16.** Shuman DI, Narang SK, Frossard P, Ortega A, Vandergheynst PJIspm. The emerging field of signal processing on graphs: Extending high-dimensional data analysis to networks and other irregular domains. 2013;30(3):83-98.

**17.** Defferrard M, Bresson X, Vandergheynst P. Convolutional Neural Networks on Graphs with Fast Localized Spectral Filtering. In: Lee DD, Sugiyama M, Luxburg UV, Guyon I, Garnett R, eds. *In: Advances in Neural Information Processing Systems.* Vol 29. La Jolla: Neural Information Processing Systems (Nips); 2016:3844-3852.

**18.** Wang J, Wang X, Xia M, Liao X, Evans A, He Y. GRETNA: a graph theoretical network analysis toolbox for imaging connectomics. *Frontiers in human neuroscience* 2015;9:386.

**19.** Watts DJ, Strogatz SH. Collective dynamics of 'small-world' networks. *Nature* Jun 4 1998;393(6684):440-442.
